# Supplementary figures and images for: COVID-19: Short term prediction model using daily incidence data
Source: PLoS One. 2021 Apr 14;16(4):e0250110. doi: 10.1371/journal.pone.0250110 (PMC8046206; doi:10.1371/journal.pone.0250110)

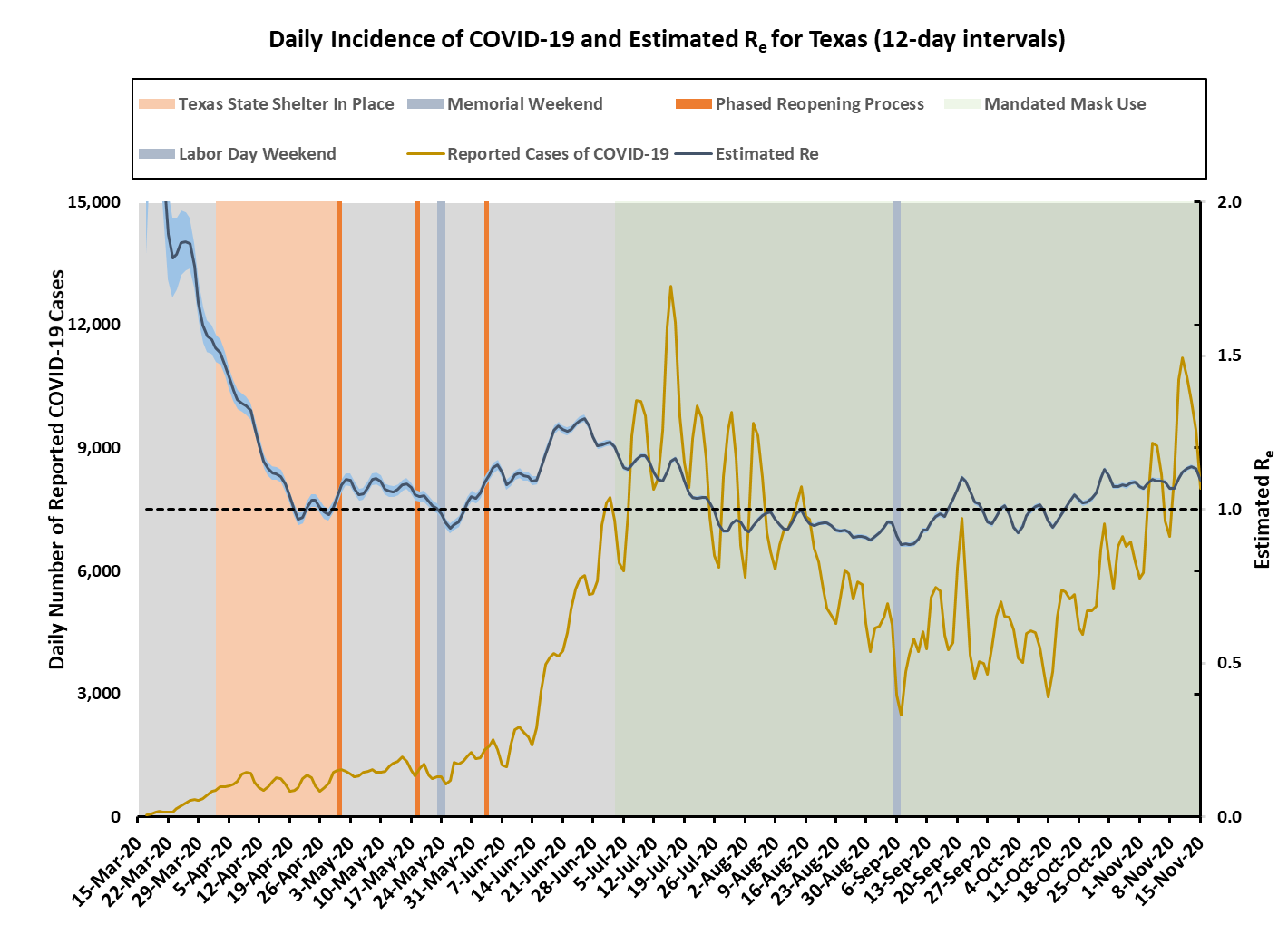

Supplement: S1 Fig — (TIF) [file pone.0250110.s002.tif]

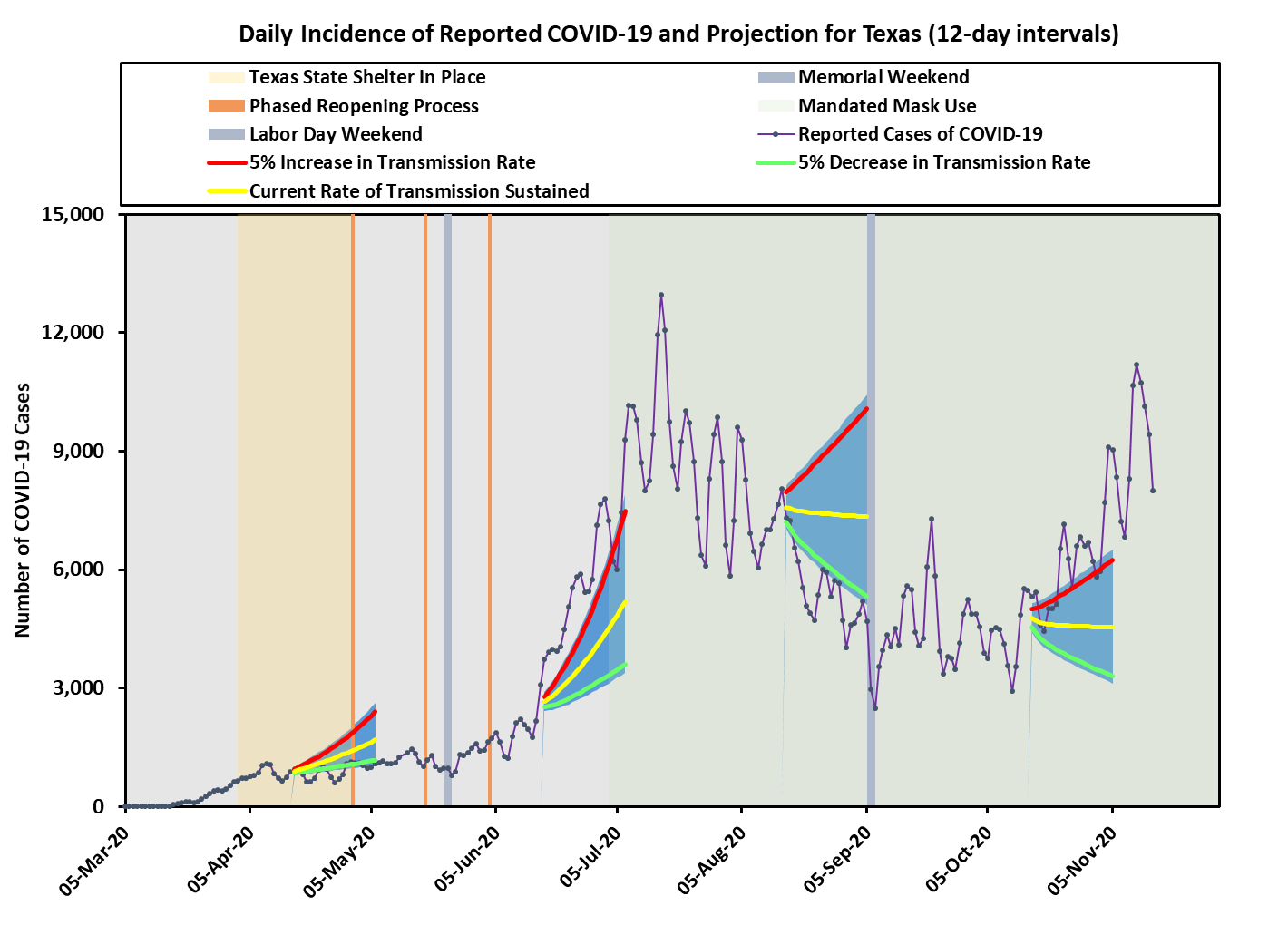

Supplement: S2 Fig — Three solid lines represent the predicted cases corresponding to current rate of transmission sustained, 5% increase in transmission rate, and 5% decrease in transmission rate. The shaded areas indicate prediction intervals. (TIF) [file pone.0250110.s003.tif]

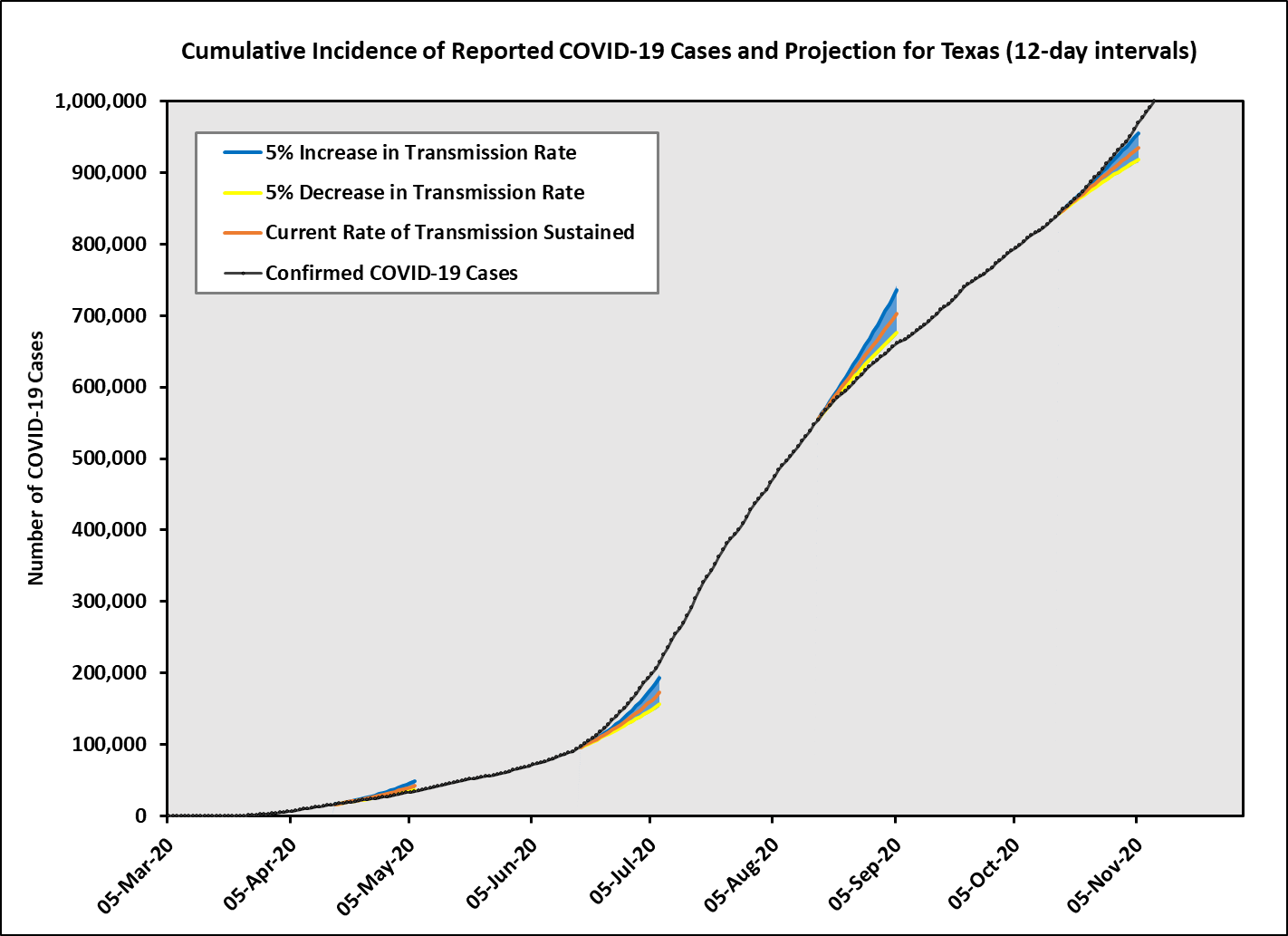

Supplement: S3 Fig — Three solid lines represent the predicted cases corresponding to current rate of transmission sustained, 5% increase in transmission rate, and 5% decrease in transmission rate. The shaded areas indicate prediction intervals. (TIF) [file pone.0250110.s004.tif]

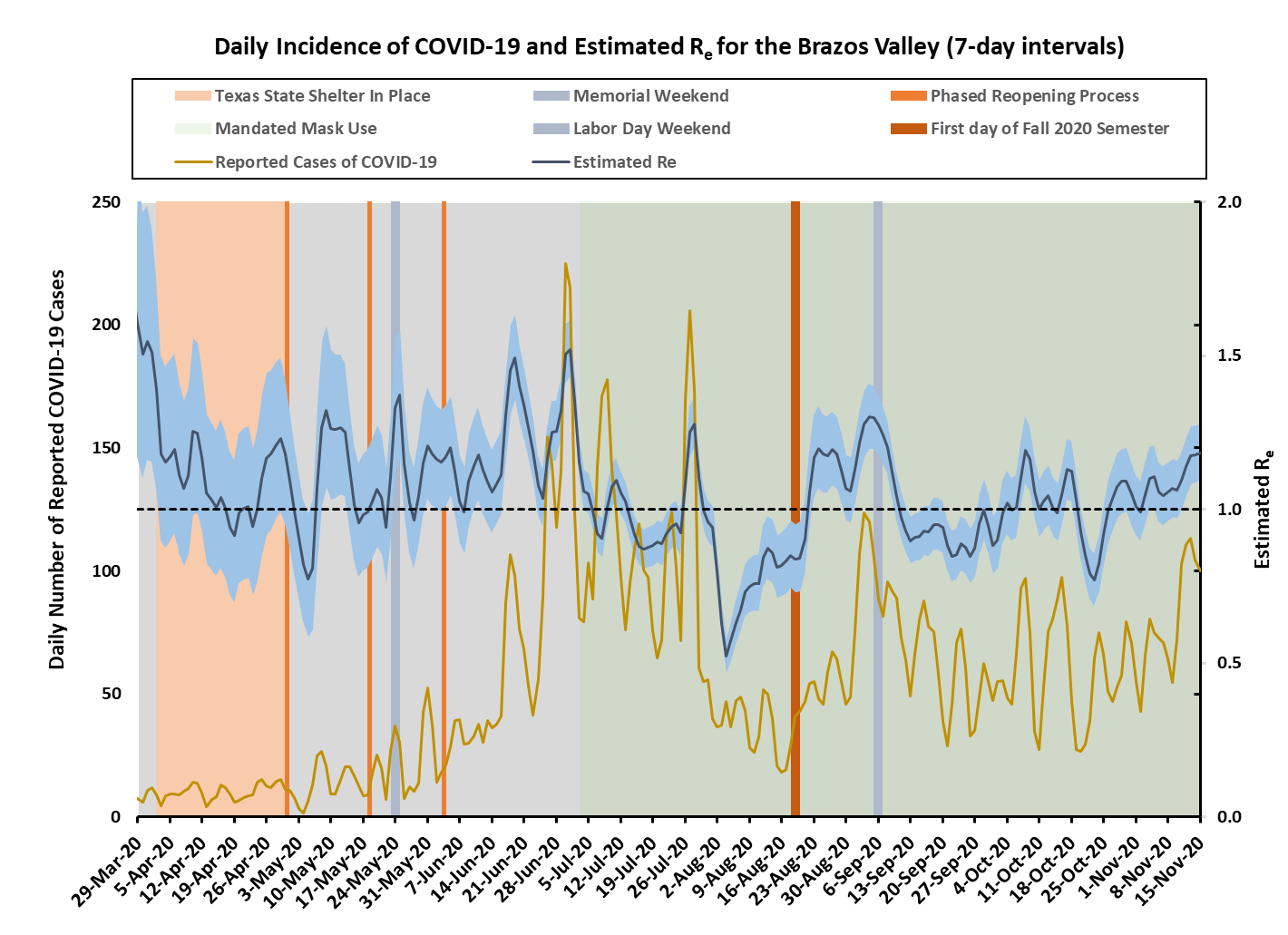

Supplement: S4 Fig — (TIF) [file pone.0250110.s005.tif]

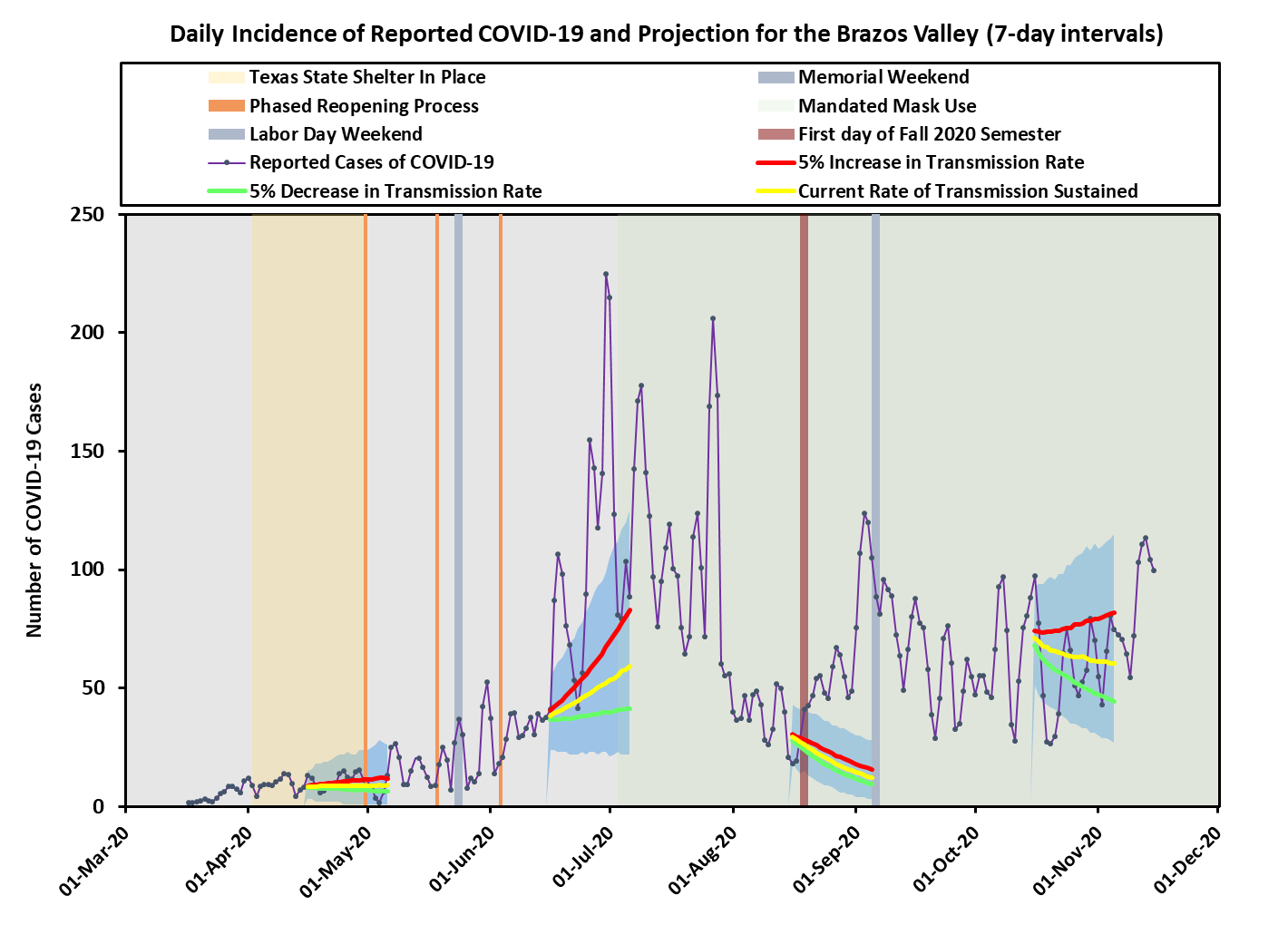

Supplement: S5 Fig — Three solid lines represent the predicted cases corresponding to current rate of transmission sustained, 5% increase in transmission rate, and 5% decrease in transmission rate. The shaded areas indicate prediction intervals. (TIF) [file pone.0250110.s006.tif]

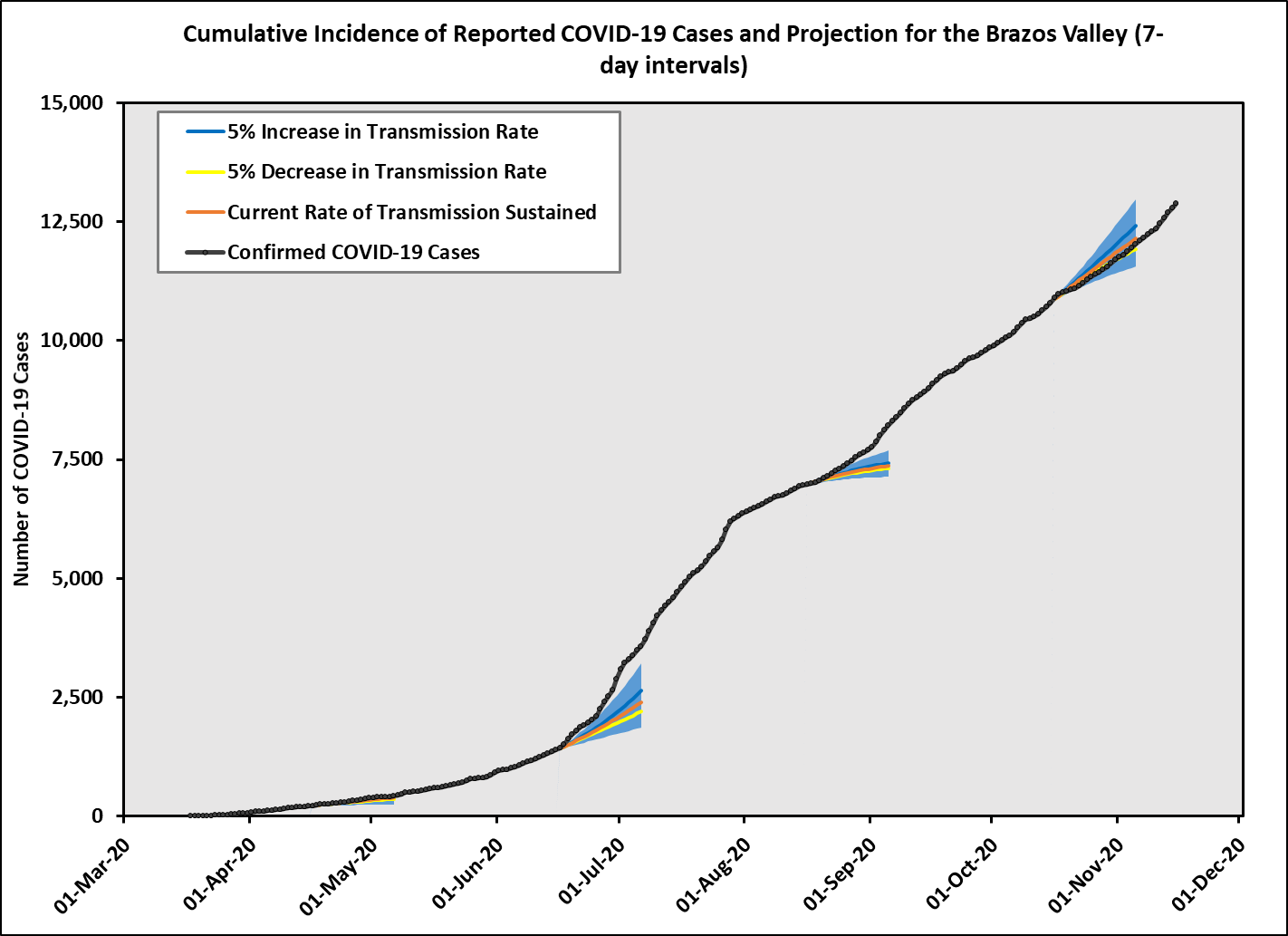

Supplement: S6 Fig — Three solid lines represent the predicted cases corresponding to current rate of transmission sustained, 5% increase in transmission rate, and 5% decrease in transmission rate. The shaded areas indicate prediction intervals. (TIF) [file pone.0250110.s007.tif]
