# Supplementary material for: COVID-19: Short term prediction model using daily incidence data
Source: PLoS One. 2021 Apr 14;16(4):e0250110. doi: 10.1371/journal.pone.0250110 (PMC8046206; doi:10.1371/journal.pone.0250110)
Supplement: Attachment — Submitted filename: Response to Reviewers 11.02.2021.docx [file pone.0250110.s008.docx]

John Schieffelin, MD

Academic Editor

PLOS ONE

February 10, 2021

Dear Dr. Schieffelin,

We thank the reviewers and the academic editor for valuable comments on our submitted manuscript entitled "COVID-19: Short term prediction model using daily incidence data" (PONE-D-20-36327). We have made changes according to your requests and replied to the questions below. We also uploaded two copies of our manuscript, one was a marked-up copy that highlighted changes made to the original version, and an unmarked version without tracked changes.

Thank you very much for your consideration. Look forward to hearing back from you soon.

Sincerely,

Hongwei Zhao

**Journal Requirements:**

1. Please ensure that your manuscript meets PLOS ONE's style requirements, including those for file naming.

Response

We have made sure that our revised manuscript meets the PLOS ONE's style requirements.

2. Please remove any funding-related text from the manuscript and let us know how you would like to update your Funding Statement. Currently, your Funding Statement reads as follows:

"The author(s) received no specific funding for this work."

Response

We have removed funding-related texts from the Acknowledgments section of our manuscript. We do not need to update our Funding Statement. Our Acknowledgments section is now as follows:

*We are appreciative of the inspiration and insight we have gotten from the Texas A&M Emergency Management Advisory Group, and Public Health Modelling Team.*

**Reviewer 1**

1. And while the authors identified a number of changes of status which could be reasonably easy to identify (and avoid) for a prediction period, they never mentioned one of the truly problematic elements related to identification of SARS-CoV2 infections (cases), which is the testing itself - not just the mentioned lag in reporting, but actual uptake of testing, and the tremendous variability that can occur in uptake of diagnostic testing, influenced by supply shortages, population interest in and access to testing, at a local or state level. The unfortunate reality is that diagnosed and reported infections with SARS-CoV2 are in fact, some unknown fraction of true infections, which also changes over time. This model gives some evidence of that, providing much tighter ranges, aligning more closely with actual case counts in the early periods, with far less precision in the late intervals.
   Response

We agree that testing poses a unique problem to the identification of SARS-CoV2 infections (cases). We added the following paragraph in the Conclusion section of our manuscript.

*A limitation for any infectious disease prediction model is the complexity inherent in how data are collected. Infectious disease reporting has long been plagued with many challenges. It is important to acknowledge that our model, as many others, relies on detection of infections through testing and reporting. In reality, the journey of a simple data element, from infection to tabulation, has many obstacles and nuances along the way. Some major complexities of the data include: policies about testing algorithms (e.g. which suspect cases are tested); if screenings or surveillance is conducted, which diagnostic test is acceptable or required for reporting; accessibility and availability of testing; administrative issues such as reporting requirements, procedures, and infrastructure. These elements can vary widely by locale and among populations within a locale. Thus, the available data are likely to represent some fraction of infections. Understanding the underlying caveats and how local situations contribute to limitations is essential to evaluating the model output. Even so, the opportunity for practical application of our model to provide insight for assessment, planning, and policy-making remains invaluable.*

**Reviewer 2**

1. Some similar results have been studied by Jin's group in Fudan (See [CCJL2020],[SZYPCC2020],[P2020]), Jin's model is well suitable for Chinese data. But the scene and data in USA are more complicated. Zhao's work is interesting.

Comment

We thank the reviewer for bringing up these references for our attention. We agree that Jin’s model is well suitable for Chinese data, but cannot be applied to USA data, since the transmission rate in USA changed frequently. We have added these references in our revised manuscript.

2. One suggestion is that we may not deal with the original number of incident cases, instead, we may consider to filter or smooth the number of incident cases, for example, 7-day average.

Response

We agree that it is better not to deal with the original number of incident cases, and instead use smoothed data series. In fact, we used a 3-day average in our original manuscript, with a weight of 0.4 for cases from current day, and a weight 0.3 for cases from the previous day and the subsequent day. We added the following paragraph at the beginning of the Method section to explain the need of the data processing step before the modeling:

*Assuming that we have observed a time series of COVID-19 incidence cases up to a time t, our goal is to make predictions of incidence cases in the next two to three weeks. In an ideal scenario, all data sets would be calibrated to the time of infection (an admitted impossibility). However, publicly available data sets most often reflect the date of reporting, which may be the date of reporting to the local health department, but more often reflects the date of reporting up the chain, such as to the State health department. As such, day-to-day variations of reported incidence cases often reflect not the true variation of the disease infection but reporting capacity. In addition, a large data dump might occur because of attempts to process backlogged data. Therefore, we propose to perform a smoothing average of data (e.g. 3-day weighted average) before performing any analysis. In the event of a big data dump, we also need to make adjustment to the data and distribute the cases over time. These adjustment to public databases would not only improve model handling but also be valuable for our interpretation and application.*
